# Spheroid Formation Enhances the Regenerative Capacity of Nucleus Pulposus Cells via Regulating N-CDH and ITGβ1 Interaction

**DOI:** 10.7150/ijbs.70903

**Published:** 2022-05-21

**Authors:** Yiyang Wang, Haoming Wang, Yunyun Zhuo, Yanzhu Hu, Xiaoxiao Li, Yanqin Xu, Biemin Sun, Min Liu, Luetao Zou, Liehua Liu, Lei Luo, Chen Zhao, Pei Li, Qiang Zhou

**Affiliations:** 1 Department of Orthopedics, The Third Affiliated Hospital of Chongqing Medical University, Chongqing 401120, China; 2 Tissue Repairing and Biotechnology Research Center, The Third Affiliated Hospital of Chongqing Medical University, Chongqing 401120, China; 3 Department of Orthopedics, Three Gorges Central Hospital of Chongqing University, Chongqing 404000, China; 4 College of Chemistry and Chemical Engineering, Chongqing University, Chongqing 400044, China

**Keywords:** Nucleus pulposus cell (NPC), extracellular matrix (ECM), tissue regeneration, microtissue, hydrogel

## Abstract

**Background:** Nucleus pulposus (NP) degeneration is the core pathological change of intervertebral disc (IVD) degenerative diseases, but currently, no effective therapy is available. With the rapid development of biomaterials and tissue engineering in recent years, biomaterial-assisted cell transplantation becomes a promising therapy for IVD degeneration. However, the application is severely limited by the weak biological characteristics of NP cells (NPCs), such as a moderate proliferation ability, weak self-renewal capacity, and minimal extracellular matrix (ECM) synthesis capacity, caused by the current inappropriate cell seeding or grafting methods.

**Methods:** Here, we developed a three-dimensional (3D) spheroidizing culture method to construct NPC spheroids and investigated repair and regeneration potential of these spheroids in vitro and in vivo. The in vitro biological characteristics (including cell viability and proliferation), and in vivo functions (including anti-degeneration potential and ability to induce tissue repair) of NPC spheroids and monolayer-cultured NPCs were compared. Furthermore, an RNA-seq-based transcriptome analysis and a series of function experiments were performed to elucidate the potential mechanisms of their differences that were involved in the tissue regeneration process.

**Results:** NPC spheroids exhibited obviously superior self-renewal and ECM synthesis capacities compared to monolayers of NPCs in vitro. In vivo, NPC spheroids generated more functional ECM components, primarily aggrecan (ACAN) and collagen type II (Col2), and markedly promoted NP regeneration in the disc degeneration model induced by partial NP excision. Additionally, the biological characteristics and functions of NPC spheroids were to some extent regulated by the interaction of N-cadherin (N-CDH) and Integrinβ1 (ITGβ1), two key mechanosensing ECM-receptors expressed on NPCs.

**Conclusions:** The NPC spheroidizing culture method is beneficial for cell renewal and the generation of functional ECM in NP tissue. The molecular mechanism involved in this regeneration process is closely associated with the regulation of the N-CDH and ITGβ1 interaction-mediated ECM homeostesis. Moreover, the strategy of hydrogel-assisted NPC spheroids transplantation may potentially be used in the future treatment of IVD degeneration.

## Introduction

Intervertebral disc (IVD) degeneration is regarded as the primary cause of low back pain (LBP), which constitutes a severe global public health problem 1. The whole IVD connects the adjacent bony vertebral bodies, and comprises three compartments: nucleus pulposus (NP), annulus fibrosus (AF), and cartilaginous endplate (CEP) 2. NP tissue contains more abundant hydrated proteoglycan (PG) molecules, primarily aggrecan (ACAN), than AF and CEP, which helps buffer compressive loading from the spine and maintain the collagen ultrastructure within the tissues 3. Thus, NP degeneration is presumed to be the initial and core pathology of IVD degeneration, and repair of the degenerated NP is particularly meaningful for IVD regeneration 4.

Current therapies for LBP, including bed rest, rehabilitation, medication, or discectomy, simply focus on symptom relief but fail to restore the homeostasis of the IVD^5^, ^6^. Notably, long-term treatment for symptom relief or discectomy might induce further inflammation of the local tissues, pain recurrence, and the occurrence of many other severe disc degeneration related diseases, such as lumbar spondylolisthesis and scoliosis 5. Therefore, therapies that re-establish both IVD structure and biological functions are urgently needed.

Biomaterial-assisted cell transplantation, a type of tissue engineering therapy, focuses on the regeneration of degenerated or damaged tissue and shows remarkable promise in regenerative medicine due to the excellent function of biomaterials in enhancing cell retention, proliferation and engraftment 7, 8. Including our previous study, many advances in biomaterials have facilitated the development of various biocompatible hydrogels to mimic the native NP extracellular matrix (ECM) structure, providing the possibility of repairing the degenerated NP 9, 10. However, the application of biomaterial-assisted cell transplantation is substantially limited by the adverse biological characteristics of NP cells (NPCs), such as a moderate proliferation ability, weak self-renewal capacity, and minimal ECM synthesis capacity 6. Additionally, immobilization of monolayer cultured cells in supporting bioscaffolds often results in low cell viability, low cell proliferation, and a heterogeneous spatial distribution 11.

The aforementioned limitations and the recent improvement in our understanding of tissue-specific microenvironments have supported the emergence of 'bottom-up' tissue engineering strategies in the form of scaffold-free multicellular modules (microtissues) and/or cell-ECM mimetic biomaterial constructs 11, 12. These approaches and strategies are more suitable for replicating the natural intricacies and modularity of human tissues or organs 13. Inspired by the 'bottom-up' approaches for tissue engineering applications, and the unique biological functions of spherical cell colonies in NP tissue 14, we constructed NPC spheroids using a three-dimensional (3D) spheroidizing culture system in the present study to replace monolayer cultured NPCs as seeded units in hydrogels and investigated repair and regeneration potential of these spheroids in vitro and in vivo.

## Materials and Methods

### Source of the human NP tissue specimens

The experimental human NP specimens used in the present study were obtained from patients (mean age: 46 years; age range: 39-54 years) who were admitted to the Spine Surgery Department of the Third Affiliated Hospital of Chongqing Medical University (CQMU). The Pfirrmann classification system was used to determine grades of IVD degeneration in preoperative MRI scans 15. Pfirrmann classification scores III-VI were defined as moderate stage of IVD degeneration, and a Pfirrmann classification score V was defined as severe IVD degeneration. Gelatinous tissue from the central region was harvested as the NP tissue. The study was approved by the Institutional Ethics Review Board of the Third Affiliated Hospital of CQMU. Informed consent was obtained from all patients for inclusion in the study. All procedures were performed in accordance with the ethical standards of the committee responsible for human experimentation and with the Declaration of Helsinki of 1975, as well as revision in 2000.

### Surgical procedure for establishing a compression-induced rat tail IVD degeneration model

Fifteen male Sprague-Dawley (SD) rats at skeletal maturity (16-20 weeks old, 500 ± 10 g body weight) obtained from the Laboratory Animal Center of CQMU were randomly divided into the control group (unloaded throughout), the 2 weeks group (compression loaded for 2 weeks), and the 4 weeks group (compression loaded for 4 weeks). Rat tail IVD levels and heights were confirmed by radiographs under inhalation anesthesia. Then, targeted rat tails were fixed with our self-developed compression loading apparatus between the 8th and 10th caudal vertebrae. As described in our previous study, an axial force from the distal side was exerted to produce 1.3 MPa of compression loading 16. All the surgical procedures of rats were performed under inhalation anesthesia. All animal procedures were performed after receiving approval from the Institutional Animal Care and Use Committee (IACUC) of CQMU.

### Surgical procedure for partial NP excision of rat tail and in situ NPCs/NPC spheroids implantation

All procedures involved in this surgery were performed with the approval of the IACUC of Chongqing Medical University. Thirty male SD rats at skeletal maturity obtained from the Laboratory Animal Center of Chongqing Medical University were randomly divided into the control group (IVDs did not undergo any surgical procedure), sham group (skin was incised and IVDs and adjacent bony vertebral bodies were exposed, but the IVD maintained its complete structure), hydrogel group (partial NP excision from IVDs and hydrogel in situ injection surgery was peformed), and hydrogel+NPCs group (partial NP excision from IVDs and hydrogel-encapsulated NPCs in situ implantation surgery), hydrogel+NPC spheroids group (IVDs exerted NP excision and hydrogel-encapsulated NPC spheroids in situ implantation surgery was performed). All the surgical procedures of rats were performed under inhalation anesthesia. According to a previous study 17, the rat tail NP was partially excised with a 21G empty needle, and approximately 50μl of hydrogel or hydrogel-encapsulated NPCs/NPC spheroids were injected into the NP cavity to fill the defect of NP tissue. Detailed descriptions of the surgical procedures were provided in Supplementary Figure S3.

### Histomorphological evaluation

Rat tail IVD (bone-disc-bone) specimens were fixed with 4% paraformaldehyde for 48 hours, and then were decalcified in 10% ethylenediaminetetraacetic acid (EDTA) for 4 weeks. The treated samples were embedded in paraffin, and central sagittal sections were cut at 6μm. After that, sections were stained with haematoxylin and eosin (H&E) and safranin O-fast green according to the manufacturer's instructions. The stained sections were observed and scanned under an optical microscope (Olympus, Japan). The histological degree of the IVD degeneration was evaluated according to the histopathology scoring system for IVD degeneration in rat models 18.

### Alcian blue staining

Alcian blue staining was performed to assess glycosaminoglycan (GAG) deposition and the distribution of the IVD samples as described in our previous study 16. Briefly, the sections were incubated in 0.2% Alcian blue solution before rinsing with deionized water. The stained sections were then observed and mounted with an optical microscope (Olympus, Japan).

### Micromagnetic resonance imaging (micro-MRI)

Micro-MRI was performed to evaluate the radiographic degree of the rat tail IVDs degeneration. The targeted rat tail IVDs were scanned with a high-resolution MRI (7.0-T MRI) using a T2-weighted imaging sequence. Degeneration scores were then evaluated in the images using the Pfirrmann classification system 15.

### Assay of the tissue GAG content

For the analysis of the sulfated GAG content in NP tissues, a quantitative assessment of GAG was performed using dimethyl methylene blue chloride (Sigma-Aldrich, USA) as described in our previous study 19. In brief, total GAG was precipitated with a 0.98 mol/L guanidinium chloride solution. Then, the optical density (OD) of each sample was detected at 595nm. The GAG contents were evaluated based on the OD value and the standard curve.

### Isolation and monolayer-culture of the NPCs

Primary rat NPCs were isolated using the method described in our previous study^16^. Briefly, the central NP tissues were carefully separated from the excisional rat tail IVDs and washed with normal saline 3 times. Next, the tissues were minced into flocculent pieces and digested with 0.2% type II collagenase (Sigma, USA) for 30 min. Then, complete medium (DMEM/F12 (Gibco, USA) containing 10% fetal bovine serum (FBS, Gibco, USA) and 1% penicillin/streptomycin (Gibco, USA)) was added to the suspension to neutralize the digestion solution. The cells were pelleted by centrifugation at 1200 rpm for 10 min and then resuspended in complete medium. Finally, the isolated primary NPCs were seeded into flasks, and incubated at 37 °C with 5% CO_2_. The cells at passage III were used for the subsequent experiments.

### Characterization of the NPCs

According to the prior studies, three major specific cell surface markers of rat NPCs (N-CDH, KRT19, and SOX9) were identified by the fluorescence activated cell sorter (FACS) using flow cytometry 20-22. Briefly, the isolated cells were trypsinized, centrifuged and resuspended in PBS. Then the cells were fixed and permeabilized by fixation and permeabilization kit, and incubated successively with primary antibodies (anti-N-cadherin (N-CDH, Proteintech, China), anti-keratin19 (KRT19, Proteintech, China), and anti-SOX9 (Abcam, USA)), and FITC-labeled secondary antibody (Abcam, USA). Finally, the expression of fluorescent intensity was quantitatively determined by the flow cytometry.

### NPC spheroid formation and observation

A three-dimensional (3D) spheroidizing culture method was used to construct NPC spheroids according to the manufacturer's instructions of microwell plates (Aggrewell^TM^, USA). Spheroid formation was scaled up via centrifugation in six-well microwell plates with initial size control by seeding an appropriate density of suspended cells (1×10^7^ cells/well). Then, the warm complete medium was added into each well to achieve a final volume as 5 ml. Approximately 50-75% of the medium was changed by the fresh complete medium per day. After 7 days of culture, NPC spheroids were harvested from the wells using 37 µm reversible strainers and 50 ml conical tubes for morphological observation under an optical microscope and subsequent functional experiments.

### Methacrylamide-modified gelatine (GelMA) hydrogel synthesis and preparation

The GelMA hydrogel was prepared using our previously reported procedure 16. Briefly, 10% (w/v) of gelatine from porcine skin (Sigma, USA) was added to PBS and incubated at 60 °C with constant stirring until the gelatine was completely dissolved. Then, methacrylic anhydride (Aladdin, China) was added dropwise to the gelatine solution within 1 h. The reaction was allowed to proceed for 4 h at 60 °C before being stopped by adding a 5× phosphate-buffered saline (PBS). Afterwards, resulting solution was sealed in a dialysis bag (Thermo Fisher, USA) and dialyzed at 60 °C for a week to remove unreacted methacrylic anhydride. Finally, the fluid was stored at -80 °C for 24 hours and lyophilized for 72 hours to produce GelMA.

### Fourier transform infrared (FTIR) spectroscopy analysis of GelMA

An FTIR spectroscopy analysis was performed to evaluate bond formation in the GelMA hydrogel according to the manufacturer's instructions. Briefly, the GelMA sample to be tested was dried and ground into powder. Afterwards, the powder was compressed in a KBr pellet and placed in a Nicolet Magna IR550 FTIR spectrometer. FTIR spectra of the samples were then analysed and recorded by the spectrometer.

### ^1^H-NMR spectroscopy analysis of GelMA

The ^1^H-NMR spectroscopy analysis was performed to evaluate the structure characterization of the GelMA hydrogel. Briefly, the lyophilized GelMA was dissolved in deuteroxide, and regulate the final concentration at 4 mg/ml. Afterwards, the solution was removed into a Bruker-600 ^1^H-NMR spectrometer. ^1^H-NMR spectra of the samples were then analysed and recorded by the spectrometer.

### Scanning electron microscope (SEM) assessment of GelMA

The microstructures of GelMA hydrogel were observed using SEM. Briefly, the lyophilized GelMA hydrogel was coated with platinum. Afterwards, the samples were moved to the plate of SEM (HITACHI, Japan), and the images were then captured at a voltage of 20 kV.

### Establishment of a hydrogel encapsulated NPCs/NPC spheroids culture network

The encapsulation procedure was performed as described in our previous study 23. Briefly, the centrifuged NPCs/NPC spheroids were mixed with the GelMA hydrogel precursor. Afterwards, the mixed suspension was pipetted into a customized polytetrafluoretyhene (PTFE) mould and exposed to ultraviolet radiation (UV) light (365 nm, 850 mW) for approximately 20 s at a distance of 8-10 cm to construct the 3D NPCs/NPC spheroids culture network.

### Cell proliferation assay

Cell Counting Kit-8 (CCK-8) assays were used to detect the proliferation of NPCs in spheroids, as described in our previous study 24. Briefly, NPC spheroids cultured for different durations (1-8 days) were collected and seeded into a 96-well plate. Afterwards, 10 ml of CCK-8 solution were added to 90 ml of culture media in each well and continuously incubated for another 2 h in the incubator at 37 °C. Finally, the optical density (OD) of the sample in each well was measured using an automicroplate reader (BD, USA).

### RNA-Seq analysis

Total RNA was extracted from the tissue using TRIzol^®^ reagent according to the manufacturer's protocol (Invitrogen, USA) and genomic DNA was removed using DNase I (TaKara, Japan). Then RNA quality was determined using 2100 Bioanalyser (Agilent, USA) and quantified using an ND-2000 spectrophotometer (NanoDrop Technologies, USA). Only high-quality RNA samples (OD260/280=1.8-2.2, OD260/230≥2.0, RIN≥6.5, 28S:18S≥1.0, and >1μg) were used to construct sequencing library. Sequencing was performed with the Illumina platform. The raw paired end reads were trimmed and quality controlled by SeqPrep (https://github.com/jstjohn/SeqPrep) and Sickle (https://github.com/najoshi/sickle) with default parameters. Then clean reads were separately aligned to reference genome with orientation mode using HISAT2 software (version: 2.1.0) 25. The mapped reads from each sample were assembled by StringTie using a reference-based approach 26. A correlation heatmap and principal component analysis (PCA) were performed with DESeq2 based on the gene expression data. The expression level of each transcript was calculated using the transcripts per million reads (TPM) method to identify DEGs (differential expression genes) between two different samples. Significantly differentially expressed genes (DEGs) (logfold change ≥1, p-adjusted < 0.05) between NPC spheroids and monolayer NPCs were assessed using DESeq2 27. In addition, functional-enrichment analysis including Gene Ontology (GO) and Kyoto Encyclopedia of Genes and Genomes (KEGG) were performed to identify GO terms and metabolic pathways in which DEGs were significantly enriched in at a Bonferroni-corrected P value ≤0.05 compared with the whole-transcriptome background 28.

### Live/Dead Assay

Cell viability of hydrogel encapsulated NPC spheroids was detected using the Live/Dead Assay Kit (Invitrogen, USA) according to the manufacturer's protocol. Briefly, the samples were incubated in the dark with 1 ml of staining fluid (1×PBS containing 4 mM EthD-1 and 2 mM calcein AM) at 37 °C for 30 min. Afterwards, the samples were imaged under a laser scanning confocal microscope (Leica, Germany) and exported as z-stacks of fluorescent images.

### Protein extraction and Western blotting

The NPCs and NPC spheroids were collected and lysed in lysis buffer (Beyotime, China) containing a mixture of protease inhibitors phenylmethanesulfonyl fluoride (PMSF, Beyotime) and phosphatase inhibitor cock-tail I (Sigma, USA). A BCA protein quantification kit was used to determine the protein concentration of each sample according to the manufacturer's protocol. Depending on the results, equivalent amounts of protein (20 μg) were loaded on the 10% or 12% SDS-polyacrylamide gel electrophoresis (SDS-PAGE) gels. After electrophoresis was completed, the separation gel was removed, and the target protein in the gel was transferred to a polyvinylidene difluoride (PVDF) membrane (Millipore, USA). After blocking with the 3% bovine serum albumin (BSA) blocking solution, the membranes were incubated with the primary and secondary antibodies, developed, fixed and exposed. The primary antibodies used in this study were as follows: anti-ACAN (1:1000; Abcam, USA), anti-Col1 (1:1000; Abcam, USA), anti-Col2 (1:1000; Abcam, USA), anti-matrix metallopeptidase-13 (MMP-13; 1:500; Proteintech, China); anti-N-CDH (1:500; Proteintech, China), anti-Integrinβ1 (ITGβ1; 1:1000; Abcam, USA), anti-Filamin A (1:1000; Abcam, USA), anti-Rac1 (1:500; Proteintech, China), anti-p-FAK 1:500; Proteintech, China), anti-Src (1:500; Proteintech, China), and anti-GAPDH (1:500; Proteintech, China).

### Immunohistochemistry (IHC) staining

IHC staining (Boster Biological Technology, China) was performed according to the manufacturer's protocol to further evaluate the matrix proteoglycan complex deposition and expression in NP tissues. Briefly, the harvested IVD samples were fixed with 4% paraformaldehyde for 48 hours, and then were decalcified in 10% EDTA for 4 weeks. Afterwards, the treated samples were sequentially fixed with paraformaldehyde for 24 h, embedded in paraffin and sectioned at 4 mm. The sections were treated with 3% H_2_O_2_ for 15 min at room temperature to eliminate endogenous peroxidase activity and incubated with 0.12% trypsin for 30 min at 37 °C to retrieve the antigen, before being blocked with normal goat serum for 15 min at room temperature. Next, the sections were incubated with primary antibodies (anti-ACAN (1:100; Abcam, USA), and anti-Col2 (1:100; Abcam, USA)) overnight at 4°C and then incubated with goat anti-rabbit IgG-HRP secondary antibody. The stained sections were photographed under a microscope (Olympus, Japan). Finally, the sections were counterstained with Harris's haematoxylin and imaged under an optical microscope. The average optical density (AOD) of five randomly selected visual fields (per immunohistochemical slice) was measured using the ImageJ analysis system.

### Immunofluorescence (IF) staining

After rehydration, tissue sections were blocked by goat serum, treated with hyaluronidase (0.8%) for 20 min at 37 °C, and then incubated with primary antibodies (anti-N-CDH (1:500; Proteintech, China), anti-ITGβ (1:1000; Abcam, USA) overnight at 4 °C. After an additional washing step, the sections were incubated with the fluorescent dye-conjugated secondary antibody (1:1000; Proteintech, China) for 2 h at room temperature. The stained sections were then labeled with DAPI and imaged under a laser scanning confocal microscope (Leica, Germany).

### Enzyme-linked immunosorbent assay (ELISA) of functional ECM content

The NPCs were seeded in the normal six-well plate and microwell plate respectively, with the density of 1×10^7^ cells/well. After 7 days culture, NPCs and NPC spheroids were collected and resuspended with PBS. After ultrasound lysis, the total amounts of ACAN and Col2 in the supernatant solution were quantitatively assessed using the ELISA kits (Abcam, USA).

### Statistical Analysis

All experiments were repeated at least three times. Data are presented as means ± standard deviations (SD). The significance of differences between each group was determined using Student's t test or one-way analysis of variance (ANOVA) based on the normality of the data. Statistical analyses were performed utilizing SPSS version 20.0 (SPSS Software, USA). All statistical charts were drawn using GraphPad Prism version 6.0 (GraphPad Software, USA) and Origin version 9.0 (Origin Software, USA). P values < 0.05 were considered statistically significant.

## Results

### Characterizations of the spheroidal cell clusters in human NP tissues

**Figure 1:**
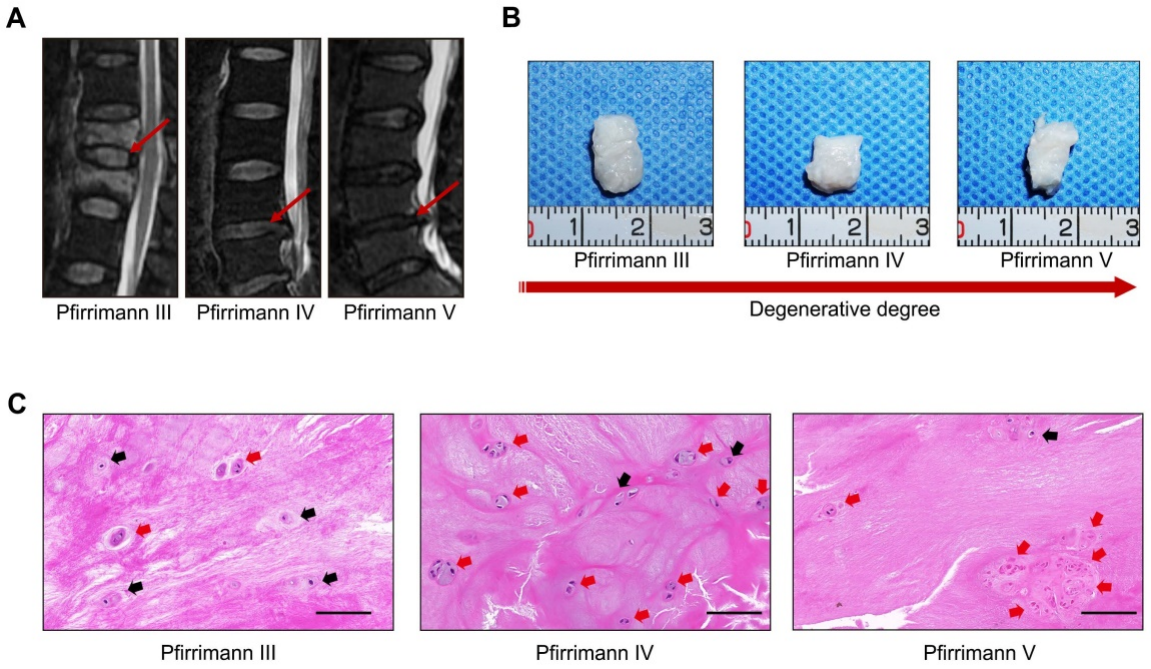
** Characterizations of the spheroidal cell clusters in human NP tissues.** (A) Representative preoperative MRI of the patients' spines with different degrees of IVD degeneration. (B) Gross view of NP samples carefully isolated from IVD surgical specimens. (C) H&E staining of paraffin sections of the NP tissue obtained from the patients' surgical IVD samples. Black scale bar = 100 μm.

Representative preoperative MRI images of the patients' spines with different degrees of IVD degeneration are presented in Figure 1A. Clinical samples of the human NP tissues were collected and classified according to the Pfirrmann grades 15. The NP samples with relatively moderate degrees of degenerative (Pfirrmann grade III-IV) were more gelatinous in texture, but the NP samples with severe degrees of degeneration (Pfirrmann grade V) exhibited varying levels of fibrotic changes according to their macroscopic morphologies (Figure 1B). As shown in the images of H&E staining (Figure 1C), cells tended to accumulate as several spherical units surrounded by abundant ECM in NP tissues. Noticeably, with the aggravation of NP degeneration, the number of NPCs decreased gradually, but most of the cells in spherical units survived. These results indicated that spherical NPC clusters exhibited stronger viability and infaust microenvironment adaptation ability.

### Characterizations of the spheroidal cell clusters in the NP tissues of rat tail IVD degeneration model

**Figure 2:**
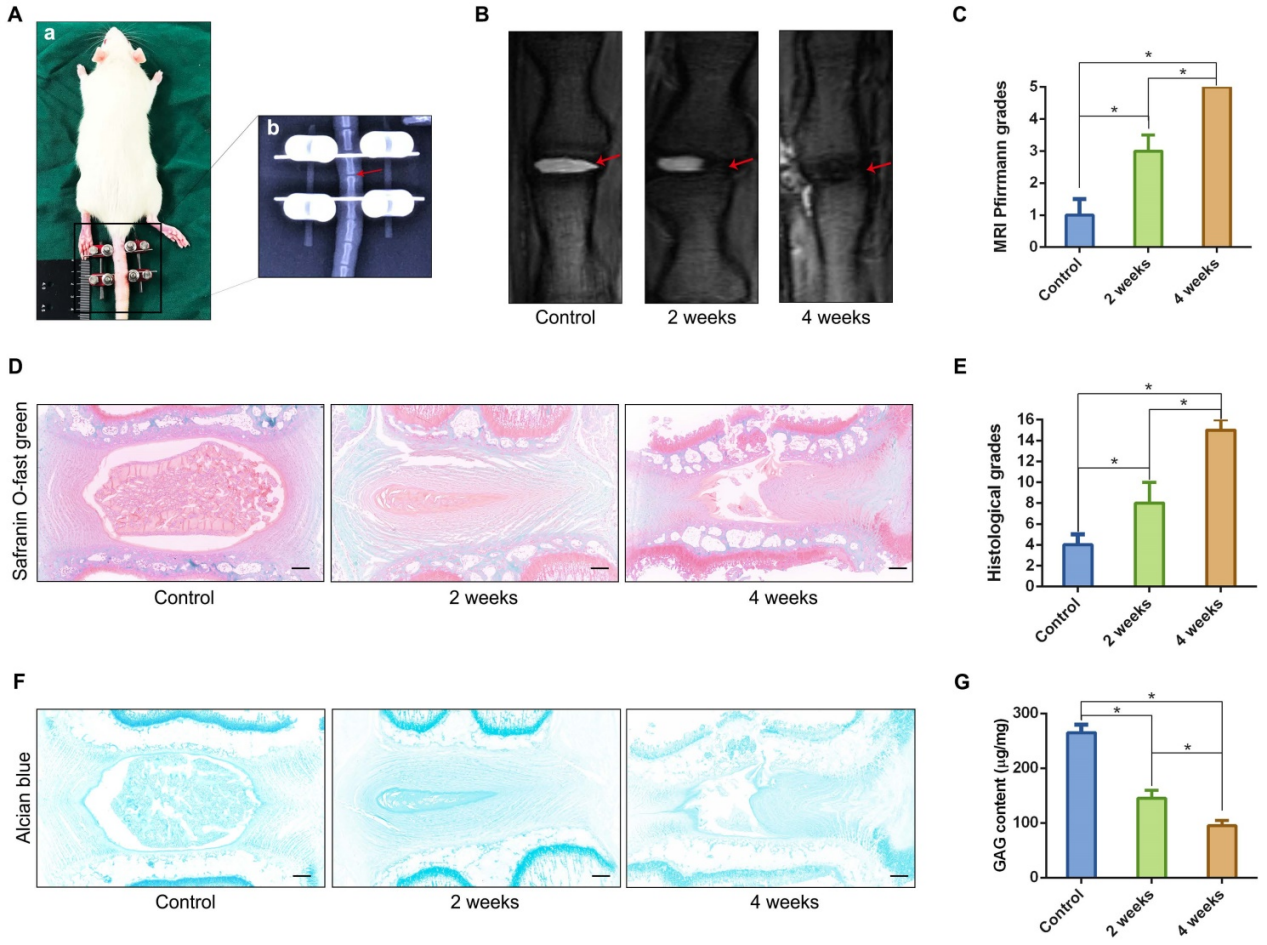
** Characterizations of the spheroidal cell clusters in the NP tissues from a rat tail IVD degeneration model.** (A) Close-up and X-ray views of the rat tail IVDs under axial compression. (B) MRI of the rat tail IVDs after different durations of axial compression. (C) Statistical analysis of changes in the Pfirrmann grade based on an analysis of MRI of the rat tail IVDs under axial compression. (D) Safranin O-fast green staining revealed histomorphological changes in the rat tail IVDs after different durations of axial compression. (E) Statistical analysis of the histological classification of the rat tail IVDs after different duration of axial compression. (F) Alcian blue staining revealed the GAG content and distribution in the rat tail IVDs after different durations of axial compression. (G) Quantification of the GAG content in NP tissues from the rat tail IVDs after different durations of axial compression. *p < 0.05, black scale bar = 200 μm.

We established a rat tail IVD overloaded compression model (Figure 2A) using the method described in our previous study 16, and verified the relationship between the classification of IVD degeneration and the number of spheroidal units to further observe the formation or disappearance of spheroidal cell clusters in the NP degeneration process. The T2-weighted MRI image of the compressed rat tail IVD indicated that the NP tissue that revealed signs of degeneration (darkened tissue signal) occurred after 2 weeks of axial compression, and deteriorated after 4 weeks of axial compression (Figure 2B-C). Safranin O-fast green staining revealed moderate and severe degenerative signs (loss of NP and increase in waviness of the fibrocartilage lamellas of AF) of the compressed rat tail IVD at 2 weeks and 4 weeks according to the rat IVD degeneration histopathology scoring system^18^ (Figure 2D-E). The GAG staining and qualification also indicated that the GAG content was gradually reduced by a prolonged duration of axial compression (Figure 2F-G). Additionally, the morphological analysis of safranin O-fast green and Alcian blue stained sections indicated that NPCs tended to aggregate and formed many dense cell clusters in IVD tissue. However, the number of NPCs decreased significantly, and several spheroidal cell units appeared to maintain the NP tissue structure during the moderate degeneration process. When disc degeneration deteriorated into the final phase, additional cell clusters disappeared and the surviving cells were isolated by fibrocartilage-like ECM in NP tissues (Figure 2D, F).

### Formation procedure and characterizations of the NPC spheroids

**Figure 3:**
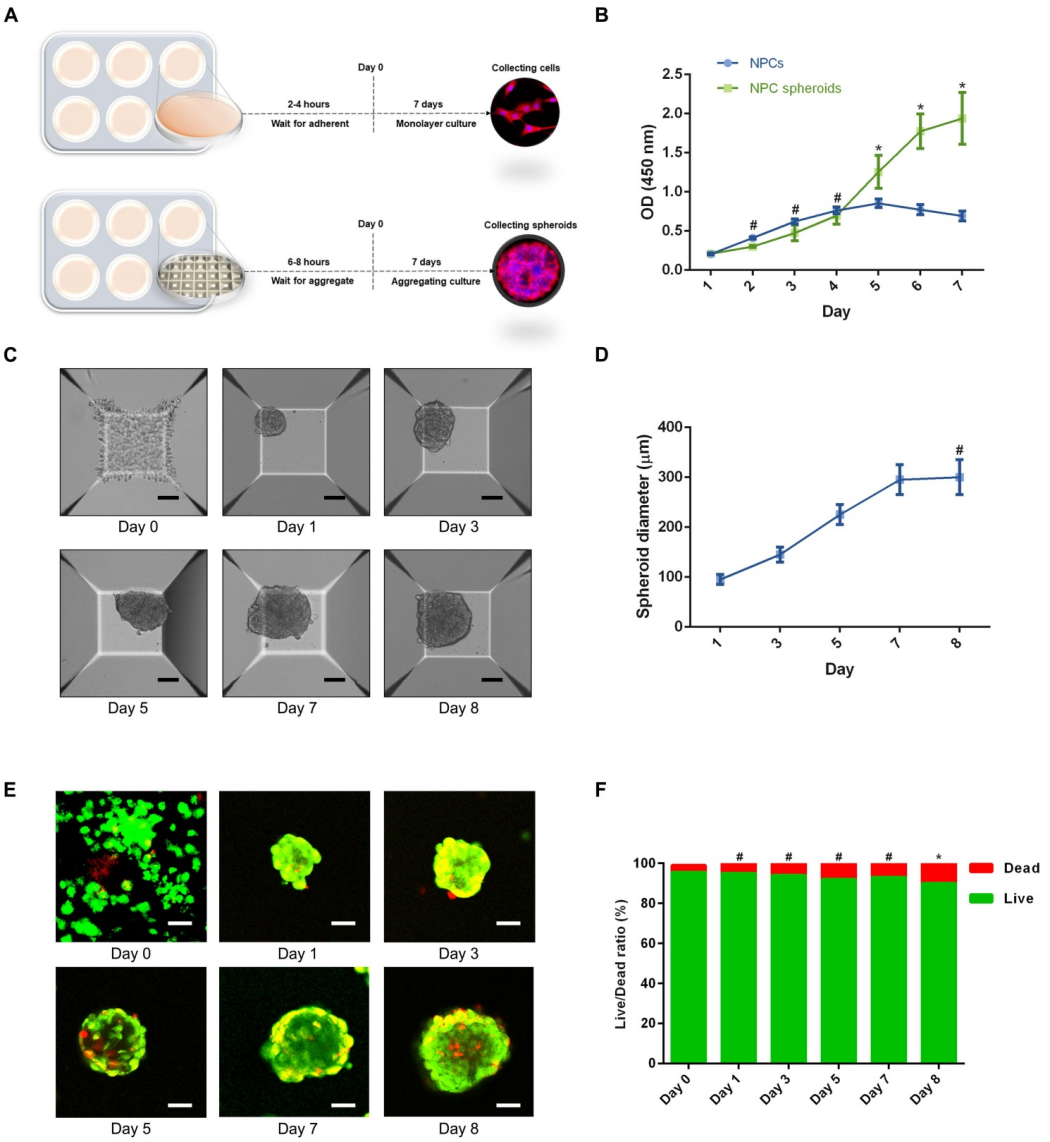
** Formation procedure and characterizations of the NPC spheroids.** (A) Schematic workflow of the NPC spheroid formation and culture procedure. (B) Changes in the viability of the NPCs under two types of culturing methods were determined using CCK-8 assay, and cell proliferation was indicated by optical density (OD) curves. (C) Optical microscopic observation of changes in the NPC spheroid size during the formation and culture process. (D) Statistical analysis of changes in the NPC spheroid diameter during the formation and culture process. (E) The viability of the cells forming NPC spheroids was analysed by fluorescent Live/Dead staining. Living cells were labelled with calcein AM, exhibiting green fluorescence. Dead cells were stained with propidium iodide (PI) showing red fluorescence. (F) Statistical analysis of the live/dead rate of the cells forming NPC spheroids. *p < 0.05 and **^#^**p > 0.05, scale bar = 100 μm.

Based on the aforementioned observations, we further established a 3D spheroidizing culture method to construct NPC spheroids, and investigated the biological characteristics of these spheroids. The phenotype identification of the isolated NPCs used in the present study was exhibited in the supplementary materials (Figure S1). As is shown in the schematic workflow of the NPC spheroids formation and culture procedure (Figure 3A), the same concentration of a single-cell suspension was seeded into normal six-well plates for monolayer culture and microwell plates for 3D spheroidizing culture. NPCs were then allowed to adhere to the bottom of a six-well plate or aggregate with each other to form spheroids in the microwell plates within 8 hours. After 7 days of culture, NPCs and NPC spheroids were harvested for further experiments. The proliferation of cells subjected to the two types of culturing methods was determined using the CCK-8 assay. As shown in Figure 3B, the proliferation speed of the monolayer cultured NPCs was faster within the initial 4 days. Afterwards, the proliferation speed of cells cultured using the monolayer method was retarded by the contact inhibition. However, the cell proliferation speed in NPC spheroids increased continuously until 7-8 days of in vitro culture. Moreover, the size of NPC spheroids increased significantly in the initial 7 days after the cells were seeded (Figure 3C-D). Subsequently, the increase in spheroid diameter slowed down and plateaued at approximately 300μm (Figure 3C-D). Additionally, we further observed the viability of NPCs forming cell spheroids by performing Live/Dead fluorescence staining. Living cells accounted for 90% of the total number of cells in spheroids (Figure 3E-F). Notably, the cell viability exhibited a slightly decrease in the group of cell spheroids cultured for 7 days compared with the initially seeded cells (Figure 3E-F).

### Viability and proliferation of the cells in GelMA hydrogel-encapsulated NPC spheroids

**Figure 4:**
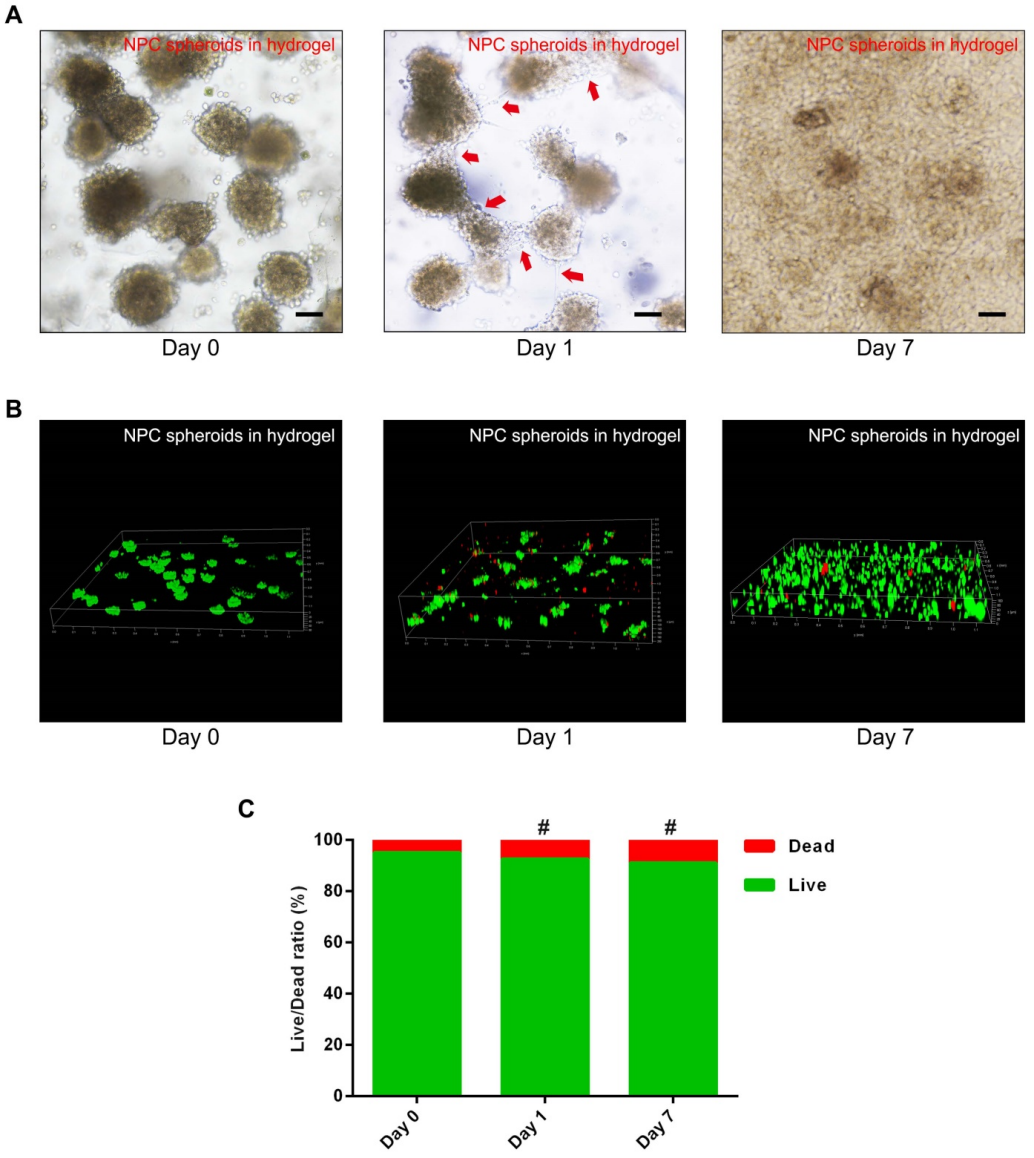
** Assessment of NPC proliferation and viability in the GelMA hydrogel.** (A) Representative optical microscopy images showed that cells in the NPC spheroids proliferated and migrated normally in the GelMA hydrogel. Red arrows represent typical signs of cell migration. (B) The z-stack fluorescent images of Live/Dead staining showed the viability of the NPC spheroids seeded in GelMA hydrogel. (C) Statistical analysis of the cell live/dead rate of the NPC spheroids seeded in GelMA hydrogel. **^#^**p > 0.05, scale bar = 100 μm.

To verify the viability and proliferation of the NPCs and NPC spheroids seeded in the GelMA hydrogel, optical microscopy observation and fluorescent Live/Dead staining were performed. The synthesis procedure and characterization of GelMA were exhibited in the supplementary materials (Figure S2). The results of fluorescent Live/Dead staining indicated that encapsulating and photocrosslinking procedure of the GelMA did not affect the viability of the NPCs seeded in the hydrogel (Figure S2). Additionally, the optical microscopic images showed that cells of the NPC spheroids proliferated and migrated normally in the GelMA hydrogel (Figure 4A). The z-stack scanning images of the fluorescent Live/Dead staining also revealed that cells of the hydrogel-encapsulated NPC spheroids proliferated normally with a high live ratio (Figure 4B-C). These results indicated that GelMA hydrogel was suitable for the encapsulation of the NPCs and NPC spheroids to construct tissue engineered NP.

### Comparison of the ECM synthesis capacity between NPCs and NPC spheroids in vitro

**Figure 5:**
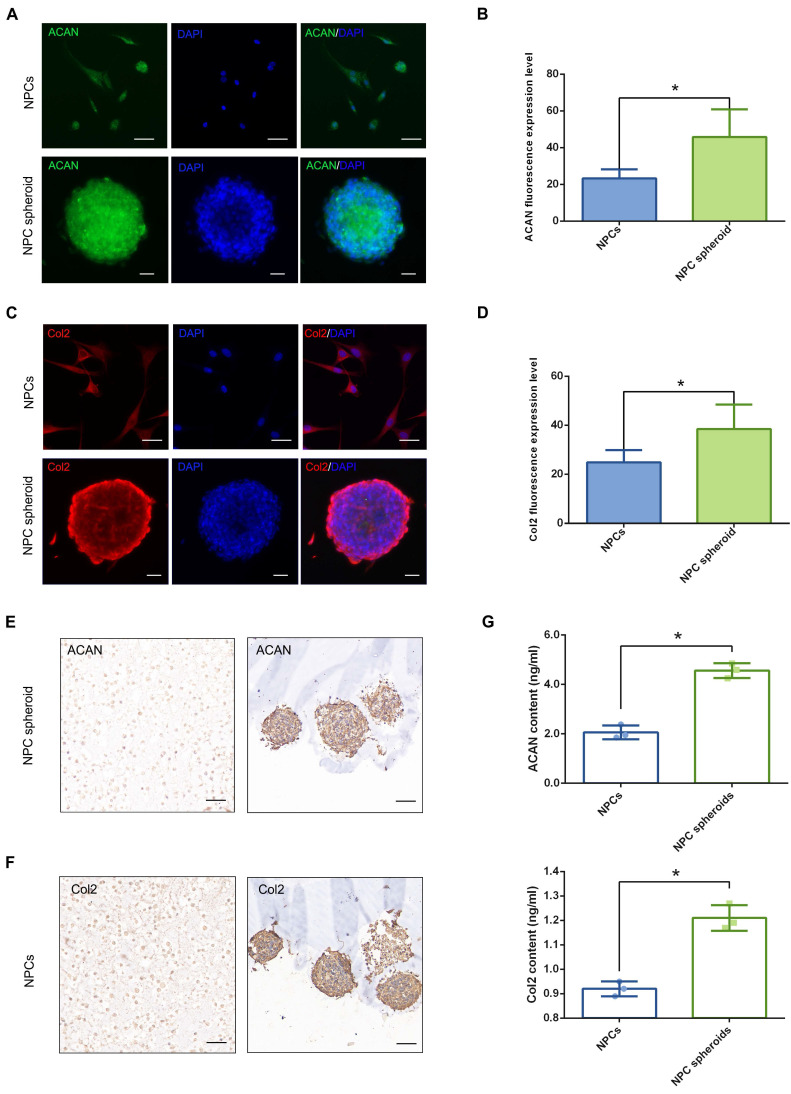
** Functional ECM synthesis capacity of the NPCs and NPC spheroids in vitro.** (A) ACAN (green) and DAPI (blue) immunofluorescence staining of the NPCs and NPC spheroids. (B) Statistical analysis of the fluorescent ACAN AOD value of the NPCs and NPC spheroids. (C) Col2 (red) and DAPI (blue) immunofluorescence staining of the NPCs and NPC spheroids. (D) Statistical analysis of the fluorescent Col2 AOD value of the NPCs and NPC spheroids. (E) ACAN IHC staining of the hydrogel-encapsulated NPCs and NPC spheroids. (F) Col2 IHC staining of the hydrogel-encapsulated NPCs and NPC spheroids. (G) ELISA quantitative analysis of ACAN and Col2 content of the NPCs and NPC spheroids. *p < 0.05, scale bar = 100 μm.

The main functional ECM components of NP include Col2 fibril and proteoglycan complexes, predominantly ACAN, which are responsible for the maintenance of the hydrophilic nature and mechanical structure of NP tissue 2. Therefore, the contents of ACAN and Col2 are important indicators to evaluate NP regeneration. The results of fluorescent staining showed that both ACAN and Col2 synthesis were markedly increased in NPC spheroids compared with monolayer-cultured NPCs (Figure 5A-D). Similarly, ACAN and Col2 expression of the hydrogel-encapsulated NPCs and NPC spheroids were assessed using IHC staining. The results revealed that expression of ACAN and Col2 were markedly enhanced in the hydrogel-encapsulated NPC spheroids compared to the hydrogel-encapsulated NPCs (Figure 5E). Additionally, the results of ELISA quantitative analysis further verified that secretion of ACAN and Col2 content were significantly increased in the NPC spheroids compared to NPCs (Figure 5G). Similarly, the Western blotting results also revealed significantly increased expression of ACAN and Col2 in NPC spheroids compared to NPCs (Figure 8E-F). Taken together, the results described above indicated that the 3D spheroidal culture method was more beneficial for the synthesis and secretion of functional NP ECM components, than the NPCs grown using the normal monolayer culture method.

### Comparison of the tissue regeneration capacity between NPCs and NPC spheroids in vivo

**Figure 6:**
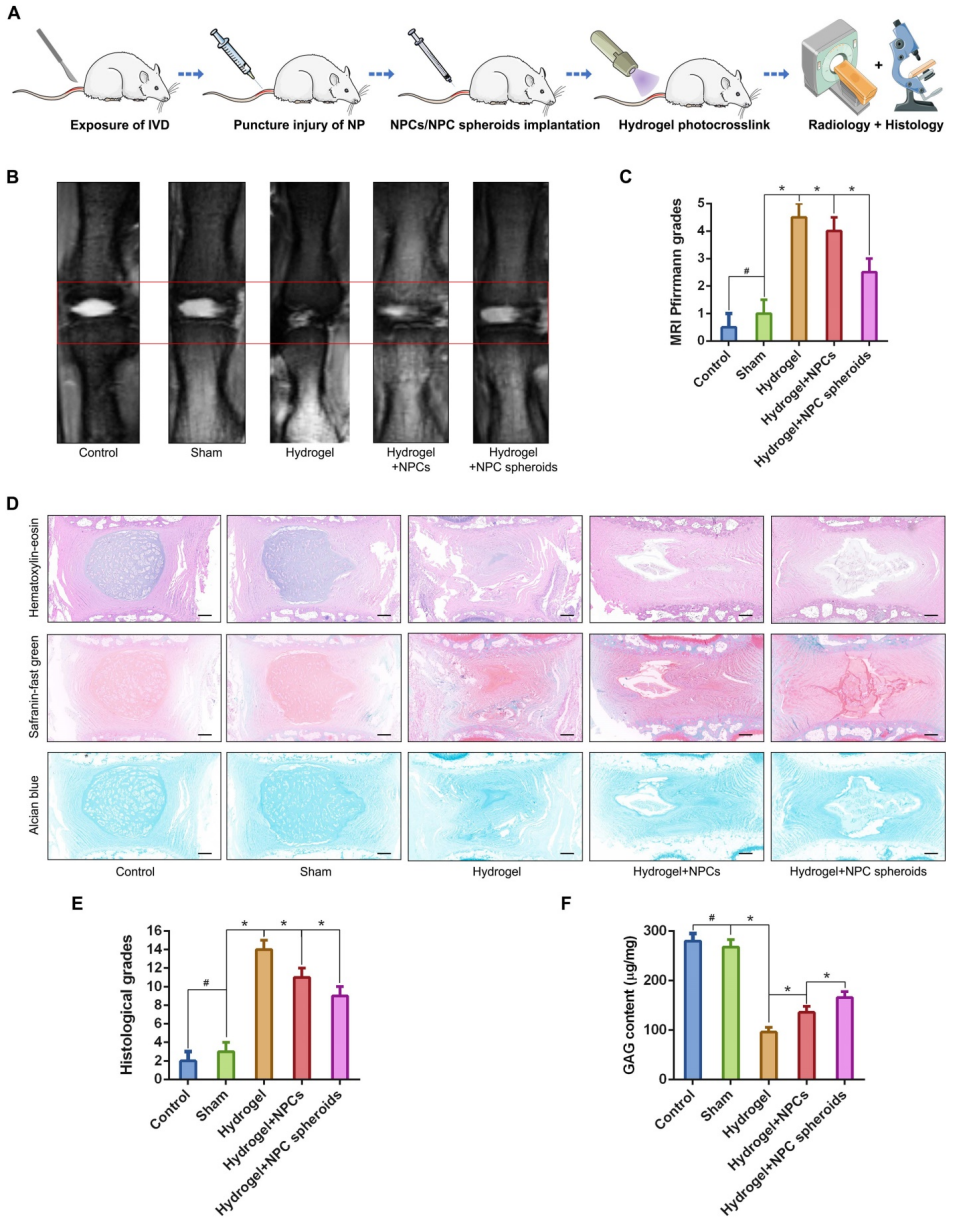
** Implantation of the hydrogel-encapsulated NPCs and NPC spheroids and evaluate of tissue regeneration capacity in vivo.** (A) Schematic illustration of the rat tail NP excision and GelMA hydrogel-encapsulated NPCs/NPC spheroids in situ implantation procedure. (B) T2-weighted MRIs of the IVD segments: the control group represents the rat tail IVDs did not undergo any surgical procedure; the sham group represents the rail tail IVDs that underwent a skin incision and exposure of IVDs and adjacent bony vertebral bodies, but the IVD maintained its complete structure; the hydrogel group represents the NP excision from rat tail IVDs and hydrogel in situ injection surgery; the hydrogel+NPCs group represents the NP excision from rat tail IVDs and hydrogel-encapsulated NPCs in situ implantation surgery; and the hydrogel+NPC spheroids group represents the NP excision from rat tail IVDs and hydrogel-encapsulated NPC spheroids in situ implantation surgery. (C) Statistical analysis of changes in the Pfirrmann grade based on MRI of the rat tail IVDs after different surgical procedures. (D) Histological assessments (including H&E, Safranin O-fast Green, and Alcian blue staining) of the IVD cell distribution and ECM structure. (E) Statistical analysis of changes in the histological degenerative classification score of the rat tail IVDs after different surgical procedures. (F) Quantification of the NP GAG content in the rat tail IVDs that received different surgical procedures. *p < 0.05 and ^#^p > 0.05, scale bar = 200 μm.

We punctured rat tail IVDs with a 21G needle to induce NP defects, which has been widely reported to be a reliable method to induce IVD degeneration^29^, ^30^, to verify the difference in tissue regeneration capacity of NPCs and NPC spheroids in vivo. NPC spheroids and monolayer cultured NPCs were then seeded in GelMA hydrogels, and the hydrogel-encapsulated NPCs or NPC spheroids were then implanted into the NP defect and cross-linked with short-time UV irradiation (Figure S3). After 4 weeks, the treated rat-tail IVDs were collected for further radiological and histological analysis (Figure 6A). GelMA is a commonly used photocrosslinking bioscaffold for cell culture that can be cross-linked by UV light (360 ± 5 nm, 850 mW) within 30 seconds at a distance of approximately 10-15cm 31. Additionally, the average aperture (approximately 200μm) of the GelMA scaffold was suitable for the migration and proliferation of NPCs (Figure S2). Radiological tests of T2-weighting MRI reflected the hydration of NP tissue, which was related to the degree of IVD degeneration. The MRI results indicated that implantation of GelMA-encapsulated NPCs and NPC spheroids alleviated NP degeneration to a certain degree, and the tissue repair effect of NPC spheroids obviously exceeded that of monolayer cultured NPCs (Figure 6B-C). However, injection of the GelMA hydrogel alone did not induce NP regeneration (Figure 6B-C). Additionally, histological observations including H&E, Safranin O-fast green, and Alcian blue staining, also indicated that the implantation of GelMA hydrogel-encapsulated NPC spheroids promoted NP repair and attenuated disc degeneration, and implantation of GelMA hydrogel-encapsulated NPCs exerted a limited NP repair effect (Figure 6D-E). However, injection of without GelMA hydrogel without cells or spheroids did not retard the degeneration process induced by partial excision of NP tissue (Figure 6D-E). As the major component of NP ECM, changes in the GAG content in the NP tissues were consistent with the results of Alcian blue staining, which jointly verified that the implantation of GelMA-encapsulated NPC spheroids was beneficial for promoting functional ECM synthesis and NP tissue regeneration (Figure 6F).

### Differences biological behaviours between monolayer cultured NPCs and 3D cultured NPC spheroids were regulated by ECM-receptor interaction pathways

**Figure 7:**
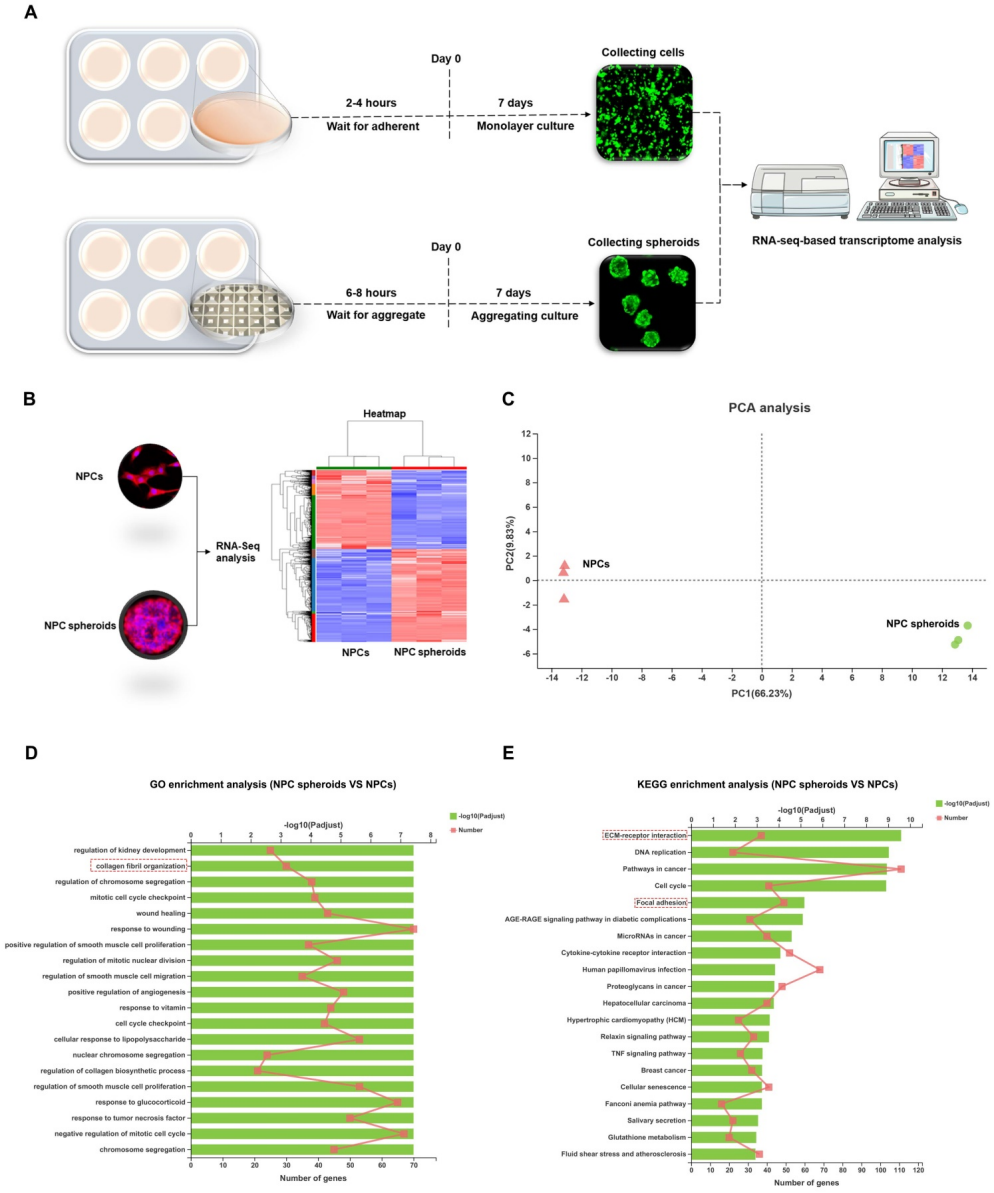
** Differences in the transcriptome between the NPC spheroids and monolayer cultured NPCs were explored using RNA sequencing analysis.** (A) Schematic workflow of the RNA-seq procedure revealing that NPC spheroids and monolayer cultured NPCs were collected after 7 days of in vitro culture for total RNA extraction, RNA-seq transcriptome library construction, DEG identification, GO and KEGG enrichment analyses. (B) Heatmap showing the upregulated and downregulated DEGs in NPC spheroids compared with monolayer NPCs. (C) PCA presenting the DEGs profiles between NPC spheroids and monolayer cultured NPCs. (D) The GO analysis showed that the upregulated DEGs in NPC spheroids were enriched in biological processes regulating collagen fibril organization. (D) The KEGG enrichment analysis showed that pathways regulating ECM-receptor interaction and focal adhesion were activated in NPC spheroids, compared with monolayer NPCs. *p < 0.05, **p < 0.01, and ***p < 0.001.

Based on the distinct biological behaviours, we further compared differences in the transcriptomes of NPC spheroids and monolayer-cultured NPCs by performing RNA-seq analysis to explore the possible mechanisms. As shown in the schematic workflow of the RNA-seq procedure (Figure 7A), NPC spheroids and monolayer cultured NPCs were harvested after 7 days of in vitro culture for total RNA extraction, RNA-seq transcriptome library construction, DEG (differentially expressed gene) identification, and functional-enrichment analyses (GO and KEGG). The statistics of upregulated and downregulated genes in NPC spheroids compared with monolayer NPCs are shown in the heatmap (Figure 7B). Principal component analyses (PCA) revealed DEG profiles between NPC spheroids and monolayer cultured NPCs (Figure 7C). Notably, according to the GO analysis, upregulated DEGs in NPC spheroids were enriched in biological processes related to the regulation of collagen fibril organization, which may explain why NPC spheroids have a superior functional ECM synthesis potential compared to monolayer NPCs (Figure 7D). Additionally, KEGG enrichment analysis revealed that several pathways highly relevant to ECM metabolism were activated in NPC spheroids, including pathways regulating ECM-receptor interaction and focal adhesion (FA) (Figure 7E).

### NPC spheroids enhanced NP functional ECM synthesis by regulating N-CDH-ITGβ1 interaction

**Figure 8:**
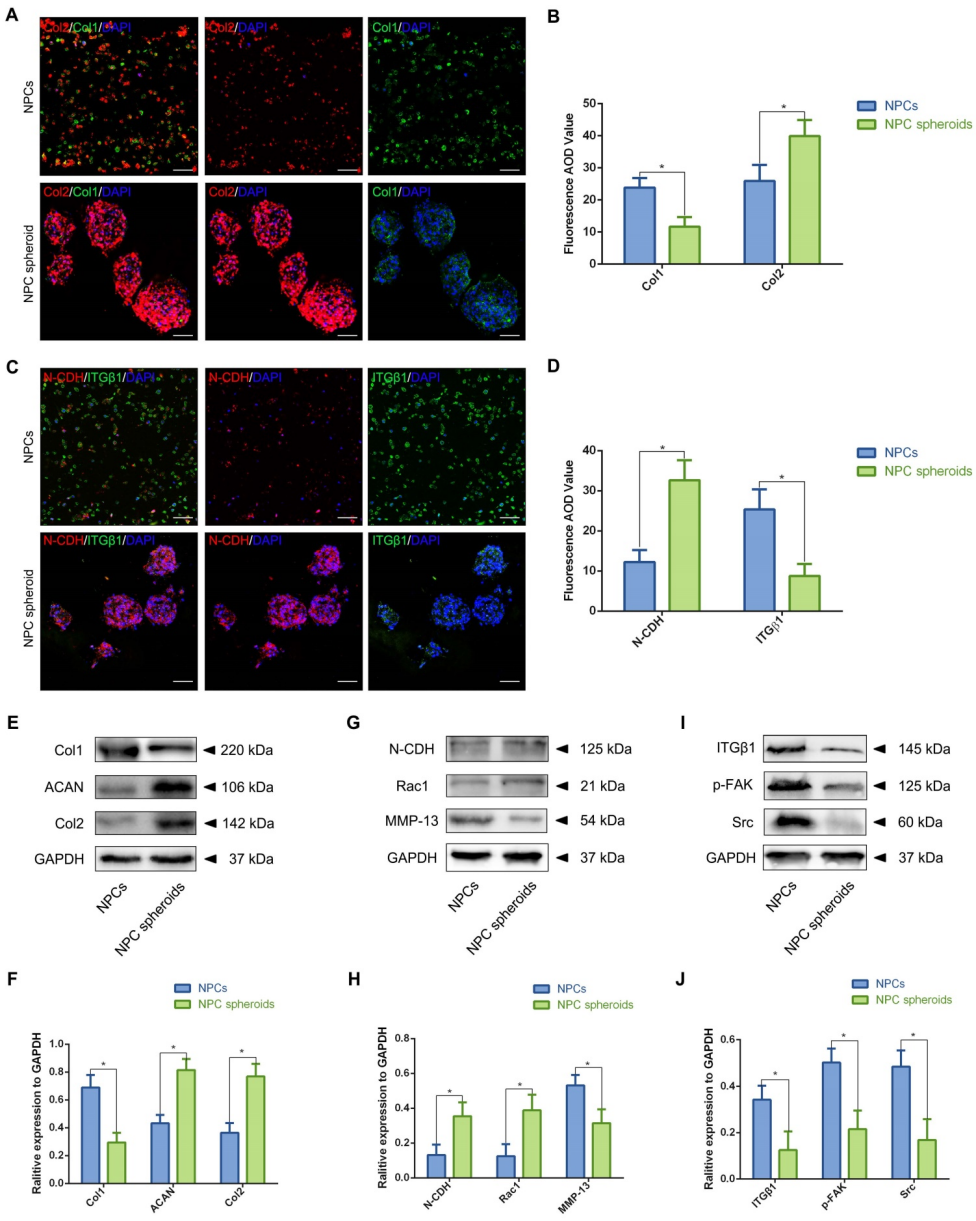
** Assessment of the N-CDH mediated AJ formation and ITGβ1 mediated FA formation of the NPCs and NPC spheroids in vitro.** (A) Col1 (green) and Col2 (red) double-labeled immunofluorescence staining of the hydrogel-encapsulated NPCs and NPC spheroids. (B) Statistical analysis of the fluorescent Col1 and Col2 AOD value of the NPCs and NPC spheroids. (C) N-CDH (red) and ITGβ1 (green) immunofluorescence staining of the hydrogel-encapsulated NPCs and NPC spheroids. (D) Statistical analysis of the fluorescent N-CDH and ITGβ1 AOD value of the NPCs and NPC spheroids. (E) Western blot analysis of the expression levels of ECM metabolism related proteins (ACAN, Col1, and Col2) in the NPCs and NPC spheroids. (F) Statistic analysis of the western blots of the ACAN, Col1, and Col2 expression levels. (G) Western blot analysis of the expression levels of AJ complex components (N-CDH and Rac1), and MMP13 in the NPCs and NPC spheroids. (H) Statistic analysis of the western blots of the N-CDH, Rac1, and MMP13 expression levels. (I) Western blot analysis of the expression levels of FA complex components (ITGβ1, p-FAK, and Src) in the NPCs and NPC spheroids. (J) Statistic analysis of the western blots of the ITGβ1, p-FAK, and Src expression levels. *p < 0.05 and ^#^p > 0.05, scale bar = 200 μm.

In order to further verify the potential regulatory mechanism involved in the process of NPC spheroids implantation-based NP regeneration, a series of testing experiments were performed. ACAN is the major proteoglycan complex in the NP matrix, and is responsible for the hydrophilic nature of NP tissue 3. In addition to the abundant content of ACAN, randomly organized collagen fibrils, predominantly Col2, also exist in the NP matrix, which forms a fibril mesh-like framework within the tissue to support other IVDs and adjacent vertebral structures 4. Thus, ACAN and Col2 act as the functional ECM components to maintain the NP matrix homeostesis. However, the ECM content changes from predominantly ACAN and Col2 to a more fibrous tissue consisting primarily of collagen type I (Col1), triggering the vicious circle between matrix stiffness and NP degeneration 32. Hence, in the present study, the expression levels of ACAN, Col1 and Col2 between NPCs and NPC spheroids were analyzed using IF staining and Western blotting. The IF staining results revealed that Col2 was expressed at markedly higher levels in hydrogel-encapsulated NPC spheroids than in hydrogel-encapsulated NPCs (Figure 8A-B), whereas the expression of Col1 markedly increased in hydrogel-encapsulated NPCs rather than in hydrogel-encapsulated NPC spheroids (Figure 8A-B). Additionally, semiquantitative analysis of ACAN, Col1, and Col2 content by Western blotting also revealed that ACAN and Col2 were expressed at higher levels in NPC spheroids than in NPCs (Figure 8E-F), whereas Col1 was expressed at higher levels in NPCs than in NPC spheroids (Figure 8E-F).

The previous studies indicated that interactions between the cadherin (CDH) and integrin (ITG) families were closely associated with disc degeneration 19, 33. Furthermore, the transcriptome analysis results in the present study also pointed out the functional role of ECM-receptor interaction-related pathways in regulating NPC spheroids biological behaviours. Therefore, we further detected the interaction of two key mechanosensing ECM-receptors (N-CDH and ITGβ1) in NP, which have been widely reported to play critical roles in regulating cell-cell or cell-ECM mechanotransduction and affect ECM anabolism and catabolism 34-36. The results of IF staining revealed that expression levels of N-CDH increased, but expression levels of ITGβ1 decreased in the hydrogel-encapsulated NPC spheroids compared to the hydrogel-encapsulated NPCs (Figure 8C-D). Additionally, semiquantitative analysis of the N-CDH-induced adherens junction (AJ) complexes (N-CDH and Rac1) content by Western blotting also revealed that N-CDH and Rac1 expressed at higher levels in NPC spheroids than in NPCs (Figure 8G-H). The ITGβ1-induced FA complexes (ITGβ1, p-FAK, and Src) content was expressed at higher levels in NPCs than in NPC spheroids (Figure 8I-J). Moreover, expression of the matrix metalloproteinase 13 (MMP13), a type of a matrix degradation enzyme associated with tissue fibrosis and disc degeneration, decreased in NPC spheroids compared to NPCs (Figure 8G-H).

### Verification of the regulatory role of N-CDH-ITGβ1 interaction in NPC spheroid-based NP regeneration

**Figure 9:**
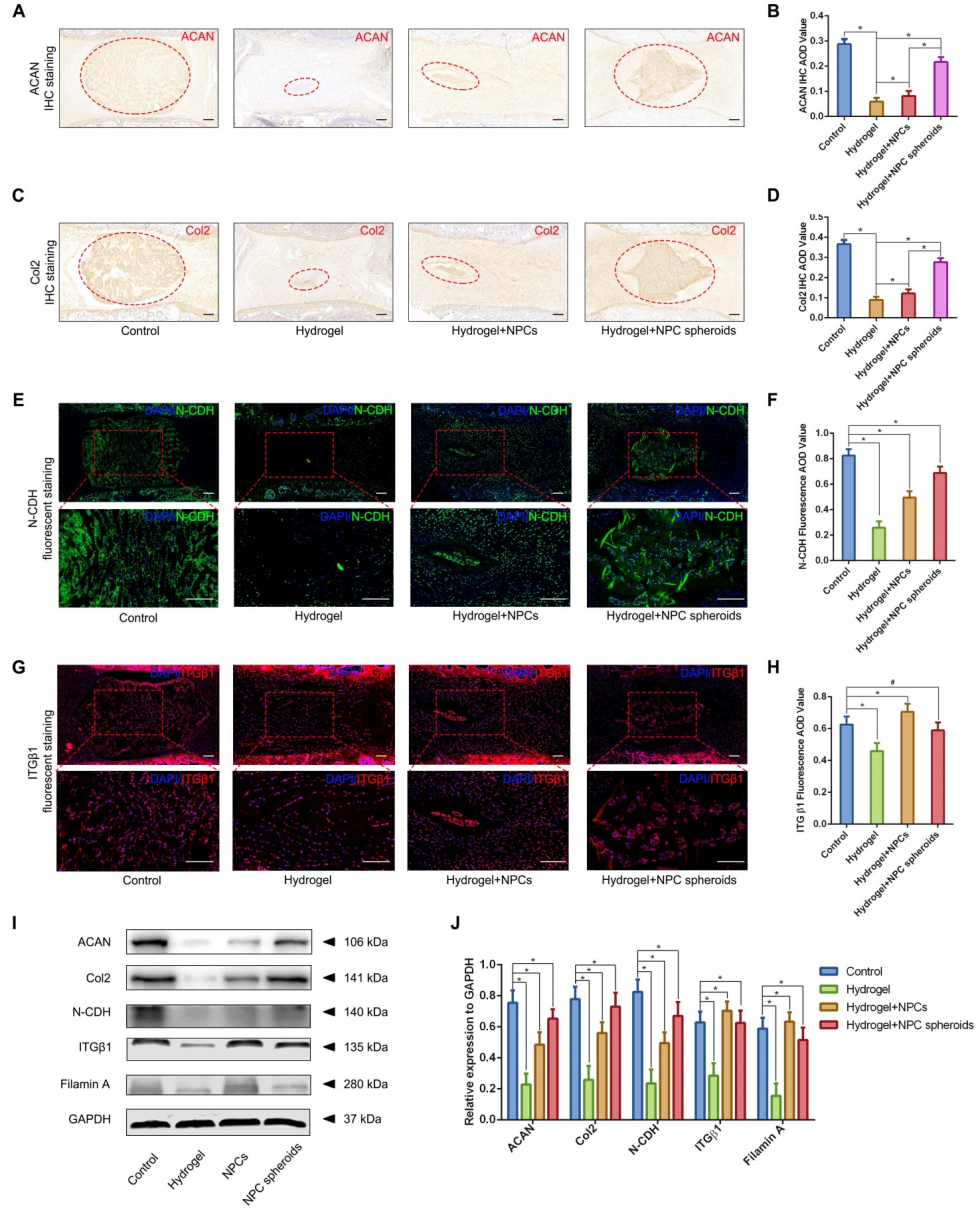
** Verification of the functional role of N-CDH-ITGβ1 interaction in regulating NPCs/NPC spheroids implantation-based NP regeneration in vivo.** (A) ACAN IHC staining of the rat tail IVDs subjected to different surgical procedures: the control group represents the rat tail IVDs did not undergo any surgical procedure; the sham group represents the rail tail IVDs with a skin incision and exposure of IVDs and adjacent bony vertebral bodies, but the IVD maintained its complete structure; the hydrogel group represents the NP excision from the rat tail IVDs and GelMA hydrogel in situ injection surgery; the hydrogel+NPCs group represents NP excision from the rat tail IVDs and GelMA hydrogel-encapsulated NPCs in situ implantation surgery; and the hydrogel+NPC spheroids group represents the NP excision from the rat tail IVDs and GelMA hydrogel-encapsulated NPC spheroids in situ implantation surgery. (B) Statistical analysis of the ACAN IHC AOD values of the rat tail IVDs after different surgical procedures. (C) Col2 IHC staining in the rat tail IVDs after different surgical procedures. (D) Statistical analysis of the Col2 IHC AOD values of the rat tail IVDs after different surgical procedures. (E) N-CDH (green) immunofluorescence staining in the rat tail IVDs after different surgical procedures. (F) Statistical analysis of the fluorescent N-CDH AOD value of the rat tail IVDs after different surgical procedures. (G) ITGβ1 (red) immunofluorescence staining of the rat tail IVDs after different surgical procedures. (H) Statistical analysis of the fluorescent ITGβ1 AOD value of the rat tail IVDs exerted different surgical procedures. (I) Western blot analysis of the levels of ACAN, Col2, N-CDH, ITGβ1, and filamin A in rat tail IVDs subjected to different surgical procedures. (J) Statistic analysis of the western blots of the rat tail IVDs exerted different surgical procedures. *p < 0.05 and ^#^p > 0.05, scale bar = 200 μm.

Based on the results of transcriptome analysis and observation of the regulatory role of N-CDH-ITGβ1 interaction on the NPC spheroids biological behaviours in vitro, we further verified the this potential mechanism in the NPC spheroids implantation-based NP regeneration process in vivo. As shown in Figure 9A-D, rat tail IVDs from all groups that underwent surgical procedures (including hydrogel injection and hydrogel-encapsulated NPCs/NPC spheroids implantation) revealed more or less shrunken positive-stained areas and lower AOD values for ACAN and Col2, than the normal control. NP tissues of the rat tail IVD subjected to GelMA hydrogel injection showed minimal ACAN and Col2 positive areas and the lowest AOD value (Figure 9A-D). ACAN and Col2 expression in NP tissues from the hydrogel-encapsulated NPCs group was slightly higher than that in the hydrogel injection group (Figure 9A-D). Notably, the expression levels of ACAN and Col2 in NP tissues from the hydrogel-encapsulated NPC spheroids group were the closest to those of the control group, although the distribution of ECM was still smaller and the tissue lacked a regular shape compared to the control group (Figure 9A-D). Moreover, Western blotting was performed to assess the expression of the ACAN and Col2 proteins, and revealed the same pattern of variation as the IHC staining results. The results of IF staining indicated decrease in N-CDH expression to different degrees in the NP tissues subjected to surgical treatments compared with the normal control (Figure 9E-F). In detail, implantation of hydrogel-encapsulated NPCs or NPC spheroids alleviated the decrease in N-CDH expression compared to the hydrogel injection group (Figure 9E-F). Moreover, N-CDH was expressed at markedly higher levels in the NP tissues from the hydrogel+NPC spheroids implantation group than in those from the hydrogel+NPCs implantation group (Figure 9E-F). However, the variation in ITGβ1 expression in each group was inconsistent with that of N-CDH. In detail, compared with the normal control group, ITGβ1 expression in NP tissues decreasd in the hydrogel injection group, but was significantly in the hydrogel+NPCs implantation group (Figure 9G-H). Notably, no significant difference in ITGβ1 expression between the control and hydrogel+NPC spheroids implantation groups (Figure 9G-H). In addition, the semiquantitative analysis of the N-CDH, ITGβ1, filamin A (downstream factor of ITGβ1) proteins by using Western blotting also revealed consistent trends for changes in their levels (Figure 9I-J).

## Discussion

The central gelatinous NP surrounded by the fibrocartilaginous AF are jointly responsible for the load bearing and buffering function of the IVD, but they are derived from different embryonic structures 2, 3. NP is derived from the notochord, while AF is derived from the somites 3. Hydrated proteoglycan molecules are more abundant in NP than AF, which helps resist the external stresses exerted on the vertebral column and maintains the collagen ultrastructure within the IVD 37, 38. In humans, IVD degeneration originates from NP tissue 37, 39. The time of the fibrocartilaginous transition of notochordal NP coincides with the appearance of the morphological signs of disc degeneration 39. Overall, re-establishment of the NP structure and biological functions is the major challenge for disc regeneration.

Under the guidance of tissue engineering strategy and biomaterial-assisted cell transplantation therapy, we previously developed an injectable tissue-engineered NP, composed of hydrogel-encapsulated NPCs, to replace degenerated NP tissue 10. Although transplantation of the tissue-engineered NP repaired degenerated NP to some extent, some deficiencies were also noted. First, in this tissue-engineered NP, the grafted NPCs are isolated by the hydrogel, which affect the viability, proliferation and migration of the seeded cells due to lack of protection provided by the natural matrix. Second, proper cell-cell communication and spatial arrangement are hindered by the bioscaffold, which influence cell physiology and attenuate functional ECM component synthesis 40, 41. In recent years, a 'bottom-up' tissue engineering strategy focusing on the construction of scaffold-free cell-rich microtissues and scaffold-based cell-biomaterial structures, has been viewed as a more suitable method for replicating the natural intricacies and modularity of human tissues or organs 11. Notably, the results of our current study revealed that spheroidal cell niches exist in both human and rodent NP tissues, and the amount of these spheroidal niches was related to the degree of disc degeneration. Additionally, previous studies also reported characteristics of the spheroidal cell cluster in NP tissue, and elucidated that these spheroidal NPC clusters revealed a stronger self-renewal capacity and ECM synthesis ability than the adhesive fibroblastic NPC clusters 14, 42. Based on the histological characteristics of NP tissue, we established NPC spheroids, a type of scaffold-free cell-rich microtissue, using a 3D spheroidizing culture plate in the current study. We found that the NPC spheroids exhibited greater cell viability, self-renewal capacity and functional ECM component synthesis ability than monolayer-cultured NPCs in vitro, indicating that NPC spheroids are more suitable seed units for constructing tissue-engineered NP.

Based on the procedure of the 'bottom-up' tissue engineering strategy, we further utilized GelMA hydrogel, a photocrosslinkable bioscaffold widely used to construct tissue-engineered organs, as the graft carrier of the NPCs/NPC spheroids. As a suitable tissue substitute, bioscaffolds should support the growth of loaded-cell and ECM deposition, and should exhibit better injectable properties for minimally invasive approaches that limit the destruction of surrounding tissues 43, 44. GelMA has been extensively used in the field of regenerative medicine, because of its injectable, biodegradable, cell-supportive, and histocompatible characteristics 45. Recent studies also reported the application of cytokine-loaded GelMA in repairing NP tissue, which it exhibited similar physical and mechanical properties to the natural NP 46, 47. The current study also verified that NPCs could proliferate and migrate normally in the GelMA hydrogel with a high live ratio, indicating GelMA hydrogel was suitable for the encapsulation of the seed cells or units to construct tissue engineered NP. However, we found that an injection of GelMA alone to fill the defect of NP tissue did not alleviate the process of NP degeneration. Due to the limited self-renewal ability of the NPCs 6 and the fast degradation speed of GelMA hydrogel 45, we speculated that the residual NPCs in situ are unable to proliferate to provide sufficient cell numbers and secret adequate functional ECM components to repair the tissue before the degradation of GelMA hydrogel. However, the implantation of hydrogel-encapsulated NPC spheroids effectively alleviated NP degeneration, as this strategy showed the closet cell distribution and ECM deposition to normal. Additionally, observations of the in vitro cultured hydrogel-encapsulated NPC spheroids also indicated that the cells in NPC spheroids migrated and proliferated normally in GelMA with excellent cell viability. Thus, our current work initially verified the availability of a NPC spheroid-based 'bottom-up' tissue engineering strategy for re-establishing NP homeostasis and retarding disc degeneration.

An RNA-seq-based transcriptome analysis was performed in the current study to further elucidate the potential mechanism underlying the different biological behaviors between the NPCs and NPC spheroids, and several pathways related to cell-cell and cell-ECM interactions were revealed. Among them, ECM-receptor interaction was the top enriched pathway that attracted our attention. Crosstalk between cell-cell and cell-ECM adhesion receptors or complexes is essential for the organization of cells into functional tissue. Healthy NP tissues contain large quantities of proteoglycans, primarily ACAN, which aggregates along the hyaluronan chains 48. The glycosaminoglycan side chains generate an osmotic swelling pressure within a randomly arranged meshwork of Col2 fibrils 49. ECM-receptor interaction-induced microenvironmental changes in matrix composition and functional component deposition are attributable to function alterations and decreased viability of NPCs 2, 4. In degenerated discs, the NP matrix stiffness increases as the tissue becomes less hydrated and more fibrous 50. As shown in our previous study, N-CDH and ITGβ1-mediated adhesive interactions influence IVD homeostasis by regulating cell mechanosensing and fate commitment 19. CDHs are regarded as the key mediator of AJ complex, structure that mainly regulates cell-cell interactions 51, while ITGs are regarded as the major mediator of FA complex, which mainly regulates cell-ECM interactions 52. Notably, it has been reported that expression of CDHs was enhanced when the cells seeded on softer medium, while expression of ITGs increased when the cells seeded on harder medium 34, 35. NPCs have a unique morphology derived from their notochordal origin, and reside in CDH type II (N-CDH) positive cell clusters in vivo 3. With the degeneration of NP, NPCs undergo phenotypic changes including decline of N-CDH expression and loss of cell clusters forming ability 20. The current study verified that spheroidizing culture method upregulated the N-CDH expression of NPCs via enhancing AJ-mediated cell-cell contact. Moreover, upregulation of N-CDH further promoted NPC clustering behaviour and NP functional ECM secretion. These results establish an important regulatory role for N-CDH-mediated cell-cell contact in preservation of NPC viability and functional ECM synthesis ability, which was consistent with the result of our current study.

Whereas, in the dense matrix microenvironment, N-CDH-mediated AJ complex become less predominant, but ITG-induced downstream pathways, which lead to the loss of NP cellularity and phenotype are activated 19, 34, 50. It has been reported that upregulation of ITG-mediated FA complex can control the strength of CDH-mediated AJ activity 36. Hence, we speculated that overexpression of ITGβ1, another key ECM mechanosensitive receptor resided in IVD tissue, could in turn impact the N-CDH-mediated NPC clustering behaviour and functional ECM secretion. In the current study, both of the in-vitro and in-vivo experiments verified that expression of the ITGβ1-mediated FA complex (ITGβ1, p-FAK, Src) markedly decreased in the NPC spheroids compared with the NPCs. Simultaneously, ACAN and Col2 expressed at higher levels in NPC spheroids than in NPCs, but Col1 expressed at higher levels in NPCs than in NPC spheroids. Changes of the NP ECM components from predominantly ACAN and Col2 to Col1, a type of collagen fibril, could induce fibrous change of tissue and increase the stiffness of ECM 32. The stiff matrix further stimulated the synthesis of ITGβ1, and triggered the vicious circle between matrix stiffness and Col1 secretion-induced NP degeneration. The potential mechanism of ITGβ1-associated FA complex enhancing Col1 secretion and tissue fibrosis is under regulated by TGFβ pathways 33, 54. Moreover, MMP 13, a type of ECM degradation proteinase associated with tissue fibrosis and NP degeneration 55, expressed at lower level in NPC spheroids than in NPCs. Based on these results, we concluded that the spheroidizing culture method enhanced N-CDH-mediated cell-cell contact, which was beneficial for NP functional ECM synthesis and tissue regeneration. At the same time, ITGβ1-induced ECM catabolism and phenotype loss were attenuated in the NPC spheroid. Thus, the NPC spheroid-based 'bottom-up' tissue engineered-NP constructing mode reveals better regenerative effect than the traditional NPC-based tissue engineering strategy.

Although some novel and meaningful findings for expanding the treatment strategies of disc degeneration are reported in the current study, several limitations also exist. First, interactions between the CDH and ITG family members are quite complicated, and are associated with cell-cell and cell-ECM mechanotransduction, adhesion formation, cytoskeletal reorganization, and intracellular signaling 34, 35, 56. The current work reports for the first time the application of an NPC spheroids grafting strategy in treating disc degeneration, and initially elucidates the potential mechanisms involved in this process. Further studies exploring the ECM-receptor interaction pathways, especially the interactions between CDHs and ITGs, should be performed in the future. Second, previous studies elucidated the effect of spheroids/microtissues on stabilizing the cell phenotype and activating cell pluripotency and differentiation, matrix-receptor interaction, focal adhesion, and hypoxia pathways, among others 57-60. However, the established studies mainly focus on the tissue repair or regeneration outcomes of spheroid/microtissue-based strategies, but the specific molecular mechanisms or regulatory pathways involved in these repair process remain controversial. Our current work initially indicated the regulatory role of ECM-receptor interactions in enhancing NPC spheroids formation and biological functions based on a transcriptome analysis. We plan to perform subsequent studies of the mechanisms of spheroid/microtissue-associated NP repair strategies.

## Conclusion

**Figure 10:**
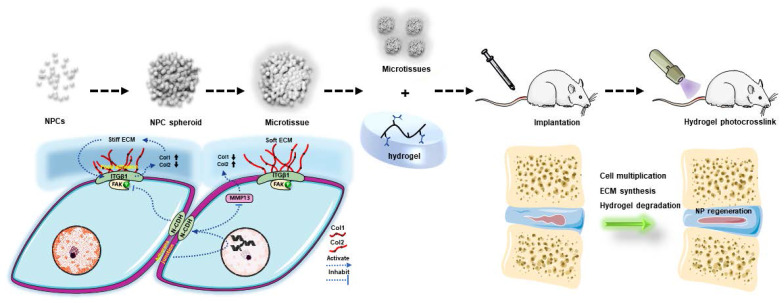
** Schematic diagram of the regulatory mechanism involved in the process of NPC spheroid-based NP regeneration.** Schematic diagram concludes the potential molecular mechanism involved in the NPC spheroids implantation-induced NP regeneration process. The 3D-spheroidizing NPC culture method enhances cell-cell adhesion, which upregulates N-CDH expression and attenuates ITGβ1 expression in NP. On the one hand, the spheroidizing culture method enhances cell-cell contact-induced N-CDH upregulation, which stimulates the secretion of components of NP functional ECM. On the other hand, the ITGβ1-mediated vicious circle between matrix stiffness and NP fibrosis is attenuated by the activity of N-CDH mediated AJ formation. At last, with the accumulation of functional ECM components and degradation of hydrogel bioscaffolds, the defect of the NP tissue is regenerated.

To conclude, the present study indicated that the NPC spheroidizing culture method is beneficial for cell renewal and the generation of functional ECM in NP tissue. The mechanism involved in this repair process is closely associated with the regulation of the interaction of N-CDH and ITGβ1, two key mechanosensing ECM-receptors expressed on NPCs (Figure 10). On the one hand, the spheroidizing culture method enhances cell-cell contact-induced N-CDH upregulation, which stimulates the secretion of components of NP functional ECM. On the other hand, ITGβ1-induced phenotype loss and tissue fibrosis are attenuated by activity of N-CDH mediated AJ formation. Moreover, the method of hydrogel-assisted NPC spheroids transplantation is proven to be a promising treatment for IVD degeneration, which expands the application prospect of microscale-based tissue engineering strategy in treating disc degenerative diseases. This study is the first to evaluate the regenerative function of NPC spheroids and initially elucidates the possible mechanism involved in this process, which helps us better understand the spheroid/microtissue based 'bottom-up' tissue engineering strategy for re-establishing IVD homeostasis and provides proof of concept for the application of this strategy in the future clinical practice.

## Supplementary Material

Supplementary figures.

## Abbreviations

IVD: intervertebral disc
LBP: low back pain
NP: nucleus pulposus
NPC: nucleus pulposus cell
AF: annulus fibrosus
CEP: cartilaginous endplate
ECM: extracellular matrix
3D: three-dimensional
ACAN: aggrecan
Col2: collagen type II
N-CDH: N-cadherin
ITGβ1: Integrinβ1
PG: proteoglycan
GAG: glycosaminoglycan
MRI: magnetic resonance imaging
GelMA: Methacrylamide-modified gelatine
UV: ultraviolet radiation
CCK-8: Cell Counting Kit-8
AOD: average optical density
OD: optical density
PCA: principal component analysis
TPM: transcripts per million reads
