# Supplementary material for: Spheroid Formation Enhances the Regenerative Capacity of Nucleus Pulposus Cells via Regulating N-CDH and ITGβ1 Interaction
Source: Int J Biol Sci. 2022 May 21;18(9):3676–96. doi: 10.7150/ijbs.70903 (PMC9254483; doi:10.7150/ijbs.70903)
Supplement: Supplementary file 1 — Supplementary figures. [file ijbsv18p3676s1.pdf]

Supplementary Materials

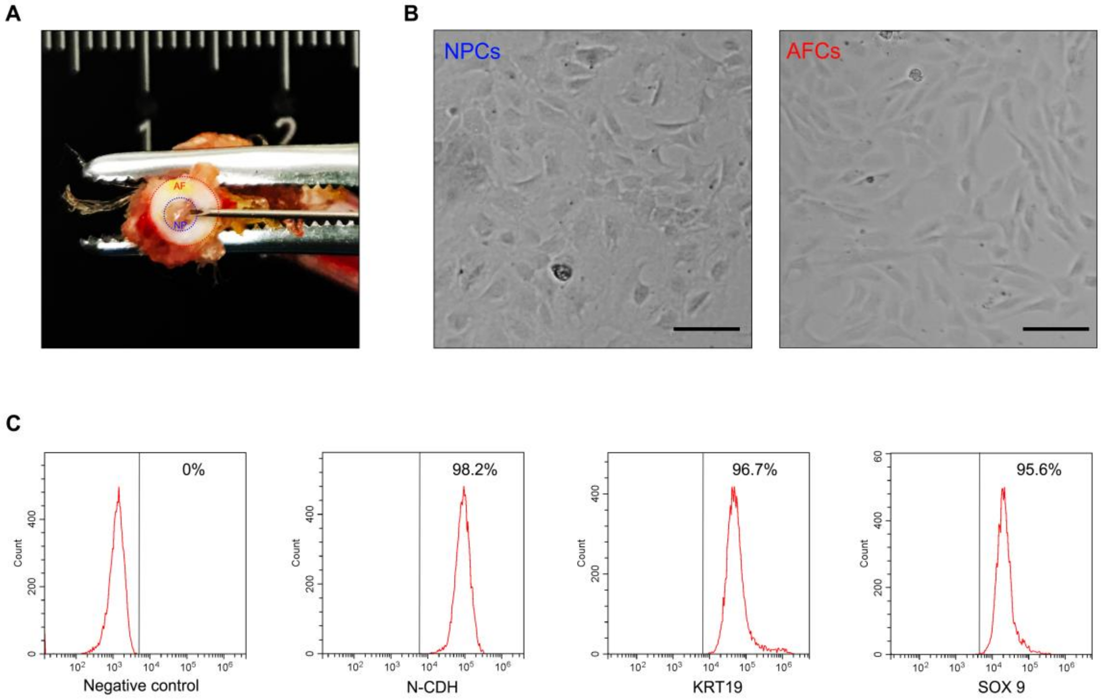

Figure S1. Characterization of the NPC morphology and phenotype. (A) Gross view of NP tissue (area inside blue circle) and AF tissue (area between red and blue circles) of the rat tail IVD. (B) Observation of the morphological differences between rat NPCs and AFCs. (C) Specific cell surface markers of rat NPCs (N-CDH, KRT19, and SOX9) were identified by the fluorescence activated cell sorter (FACS) using flow cytometry. Black scale bar = 100 μm.

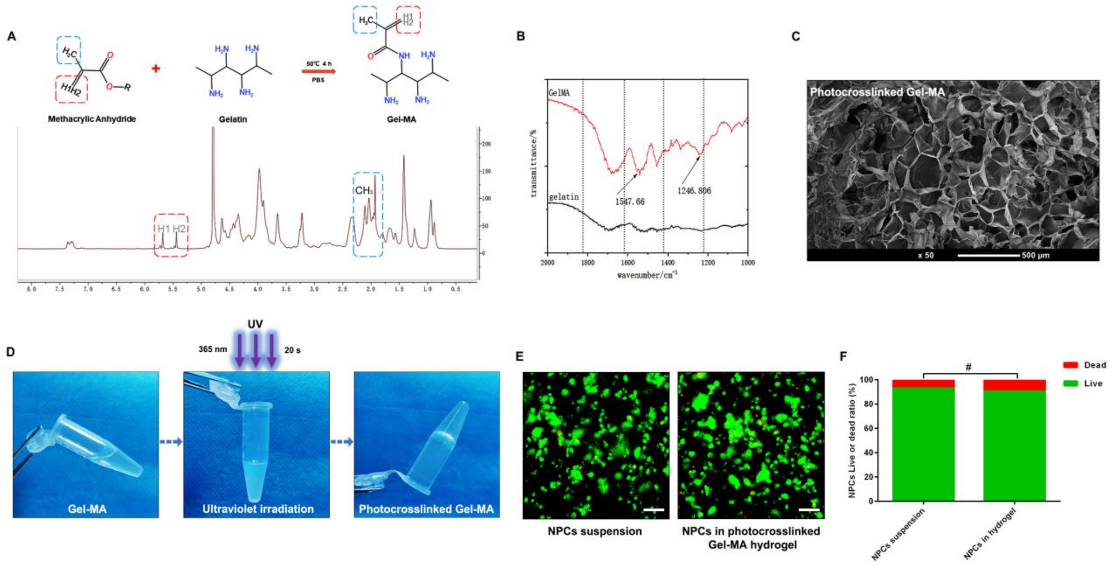

Figure S2. Characterization and chemical bond formation in the GelMA hydrogel. (A) Schematic diagram of the GelMA chemosynthesis process and ^{1}H-NMR spectroscopy analysis of the GelMA chemical structure. (B) Chemical bonding formation in GelMA was analysed by Fourier transform infrared (FTIR) spectrometer: the hydrogen bonding force in gelatine was weakened due to the migration of characteristic absorption peaks at 1547.66 cm^{-1} (representing parahelium N-H bonds) and 1246.806 cm^{-1} (representing basic nitril N-H bonds), which indicated that methacrylate anhydride was successfully grafted to gelatine. (C) SEM observation of the GelMA microstructure. (D) Photographs showing the rapid gelation of the GelMA hydrogel within 20 seconds of UV irradiation. (E) The viability of the NPCs seeded in GelMA hydrogel was analysed by fluorescent Live/Dead staining. (F) Statistical analysis of the live(green)/dead(red) rate of the hydrogel-encapsulated cells. *****$p < 0.05$ and $^{\#}p > 0.05$, scale bar = 100 μm.

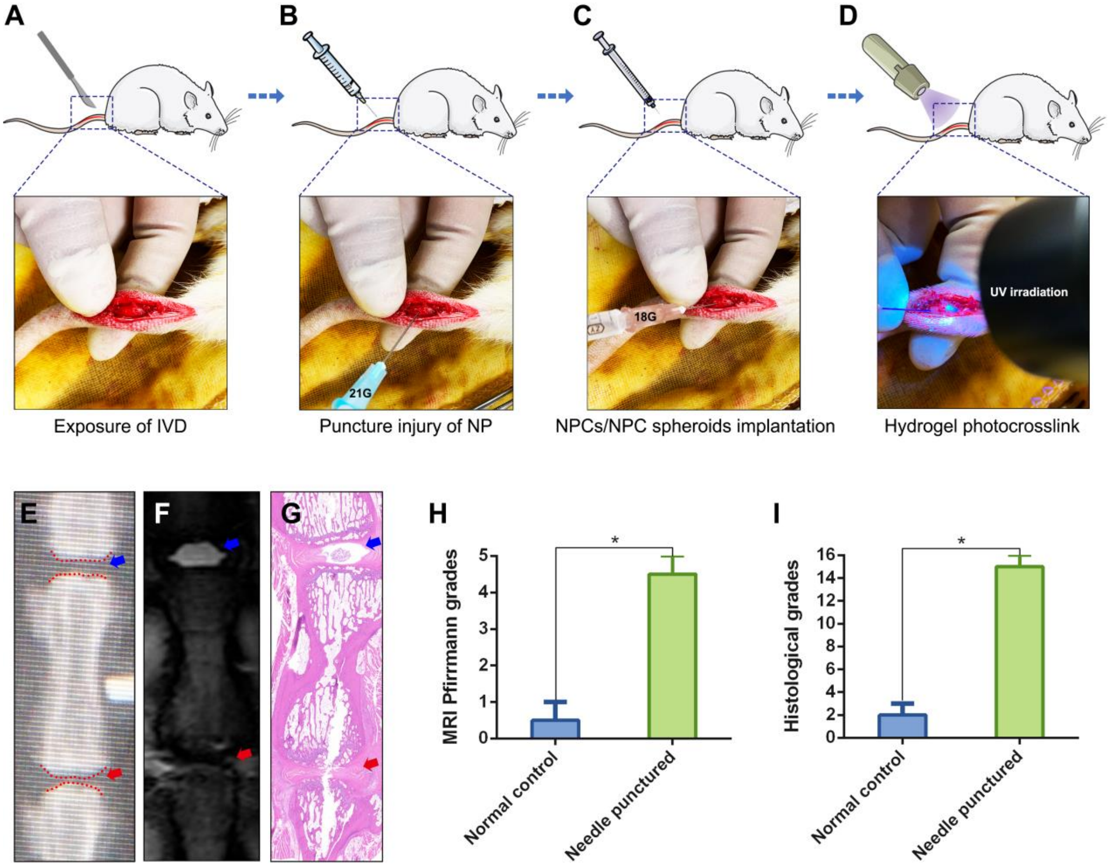

Figure S3. Schematic illustration and representative images of the surgical procedure. (A) Incision of the skin and exposure of IVDs and adjacent bony vertebral bodies. (B) Puncture of the IVD by a 21 G needle to induce NP defects. (C) In situ injection of 50μl cell-free GelMA hydrogel or GelMA hydrogel-encapsulated NPCs/NPC spheroids with an 18 G needle. (D) Photocrosslink of the GelMA hydrogel by 20 s of UV irradiation. (E) X-ray views of the rat tail IVDs (normal control: the upper segment; puncture injured group: the inferior segment) at 4 weeks after surgery. (F) MRI of the rat tail IVDs at 4 weeks after surgery. (G) H&E staining revealed histomorphological changes in the rat tail IVDs at 4 weeks after surgery. (H) Statistical analysis of changes in the Pfirrmann grade based on an analysis of MRI of the rat tail IVDs. (I) Statistical analysis of the histological classification of the rat tail IVDs. Blue arrows represent the normal control IVD segment. Red arrows represent the needle puncture injured IVD segment.
